# Imposter Syndrome Among Pre-service Educators and the Importance of Emotion Regulation

**DOI:** 10.3389/fpsyg.2022.838575

**Published:** 2022-06-30

**Authors:** Matthew LaPalme, Peihao Luo, Christina Cipriano, Marc Brackett

**Affiliations:** Yale Center for Emotional Intelligence, Yale University, New Haven, CT, United States

**Keywords:** teacher research, wellbeing, minorities, race, gender, emotion regulation

## Abstract

This study examined the prevalence and impact of imposter syndrome (IS) on a sample of pre-service educators. We report a majority of pre-service educators experience IS; 93% experience moderate levels and 54% had frequent or severe levels of imposter thoughts, and further that IS was negatively associated with educator well-being. We also investigated the effects of minority group membership on experiences of IS, and found that IS was more severe for women and queer minorities, but less severe for racial minorities. Lastly, we investigated the potential for healthy emotion regulation to mitigate the effects of IS on pre-service educator well-being and found that adaptive emotion regulation strategy use mitigates the effects of IS, which may provide a viable means for addressing this pervasive issue among educators, and specifically among those with minoritized identities. We discuss the implications of our findings for educational training and improving the experiences of pre-service educators.

## Introduction

The teaching profession faces systemic shortages of new educators, with more than 11% of incoming educators leaving the profession after the first year on the job and 44% leaving the profession within 5 years (Ingersoll, 2002; Ingersoll et al., 2018). Rates of burnout among educators, often considered the primary reason why they leave the profession, are highest for educators from minoritized groups (Carver-Thomas, 2018) with research showing a widening gap in their attrition (Ingersoll et al., 2017). An important, understudied, and malleable factor that contributes to these high rates of burnout, especially those from minoritized groups (Innstrand et al., 2011; Peteet et al., 2015; Viehl et al., 2017), is imposter syndrome (Villwock et al., 2016).

Imposter syndrome (IS) is the belief that one’s success is due to luck and that others will discover your lack of competence (Clance, 1985). Educators who experience IS fear evaluation, believing that others have overestimated their abilities (Clance, 1985; Kolligian and Sternberg, 1991), experience difficulties accepting praise, and often discount their successes as accidents or errors (Harvey and Katz, 1985). Although IS has demonstrated associations with a host of negative outcomes in adulthood, such as psychological depletion and mental distress (Henning et al., 1998; Hutchins and Rainbolt, 2016; McClain et al., 2016), prior research provides hope that we can address and mitigate IS through the promotion of healthy emotion regulation and coping skills (Naragon-Gainey et al., 2017; Patzak et al., 2017; Hutchins et al., 2018).

Feelings of being an imposter may be a widespread psychological phenomenon among educators, but little is known about how and for whom IS may emerge during the transition to a career in education. Pre-service educators transitioning into the teaching profession face substantial hurdles to success (e.g., high emotional labor demands, work overload, and classroom management difficulty; Fogarty and Yarrow, 1994; Vesely et al., 2014; Paquette and Rieg, 2016) that may increase their stress and likelihood of feeling like an imposter (Sims and Cassidy, 2019). IS can be pernicious in that educators with specific identities may be more likely to develop persistent imposter syndrome than others. For example, studies examining the effects of this phenomenon among women find that substantial proportions experience imposter thoughts (Clance and O’Toole, 1987; Parkman, 2016; Ladge et al., 2019). Other studies have found varied differences in the prevalence of IS when comparing racial minority and majority groups (see Bravata et al., 2020, for a review), including the extent to which racial minority group membership may predispose or be a risk factor for early-career educators in developing imposter syndrome.

The present study examines the prevalence and impact of IS among a large and diverse group of pre-service educators at Teach for America (TFA) to better understand how imposter syndrome may negatively influence teacher well-being. Teach for America is a national non-profit organization that develops educators to work in underserved communities across the United States. TFA corps members, unlike other educators, join TFA without necessarily having prior teaching experience and receive 5 weeks of intense classroom training and preparation. Because most of the corps members joining TFA are new to the teaching profession, this population is ideal to examine how imposter syndrome may influence the experience of pre-service educators during their transition into their roles. There are differences however, between TFA corps members and the population of educators in general, which we note here. TFA corps members tend to be more diverse compared to educators in the US in general. Notably, TFA educators are more racially diverse whereas educators generally tend to be White (81% of educators; NCES); our sample also contained notable representation of queer educators (educators identifying as belonging to a non-normative gender identity or sexual orientation). TFA educators also tend to be younger (average age is 25 years old) whereas the average age of teachers in US public schools in general is 43 years old. TFA corps members also tend to be recruited from pools of high performing elite graduates and are often assigned to work in underserved communities (Maier, 2012). At the outset of their careers, research has also noted that TFA corps members view teaching as less of a long-term commitment (Labaree, 2010) compared to educators who enter the profession through more traditional routes. Finally, we note that research finds that TFA educators are just as effective as other educators (Glazerman et al., 2006) and may even have net positive effect on student math achievement (Glazerman et al., 2006; Backes et al., 2019). This is important to contextualize as it points to high imposter syndrome being a product or phenomenon of entering the teaching profession, rather than as a function of competence among TFA educators.

Using this sample, we also explore the associations of gender, race, and queer identity to determine which groups, if any, are more vulnerable to the effects of imposter syndrome early in their career. We draw on a relational demography perspective and theorize that educators belonging to a minoritized demographic group will experience more imposter syndrome. Finally, we examine the extent to which emotional intelligence may benefit pre-service educators in restoring well-being and reducing reported feelings of IS. Prior research has demonstrated the capacity of EI to buffer against negative experiences (Szczygiel and Mikolajczak, 2018), and we explore this as an avenue to mitigate the pervasive phenomenon of IS.

### What Is the Prevalence of Imposter Syndrome Among Pre-service Educators?

IS among pre-service educators is a largely underexplored area of research. Numerous studies have shown that IS is commonplace among education professionals at both the K-12 and post-secondary level (Clance and O’Toole, 1987; Knights and Clarke, 2014), and more recent research is emerging on the extent to which early-career educators experience IS (Sims and Cassidy, 2019), with findings that they experience levels of IS equal to or higher than more experienced educators, in general. However, prior literature has yet to examine the extent to which educators entering the teaching profession may experience persistent imposter thoughts. Literature examining pre-service teachers has primarily sought to investigate the importance of teacher credentials and teacher educational attainment and achievement — which have relatively small relationships with teacher success (Clotfelter et al., 2007; Harris and Sass, 2011; Vagi et al., 2019). To address this gap, the present study seeks to understand the extent to which IS may be prevalent among pre-service educators.

Educators entering the teaching profession are particularly susceptible to multiple stressors, including time management and heavy workloads (Fogarty and Yarrow, 1994; Vesely et al., 2014; Paquette and Rieg, 2016). New educators must also fill competing roles of teacher and learner which can present challenges to educators’ psychosocial health. For example, many teachers fulfill their education certification requirements at the school, district, and state level when they begin being their careers as the instructional leads in their classrooms. In addition, 99% of teachers report engaging in time-consuming professional development activities (Rotermund et al., 2017). These work-role conflicts limit time for self-care, are negatively associated well-being, and can lead to self-doubt about desire or capability to teach (Miller and Flint-Stipp, 2019). New teachers also tend to report greater difficulty in managing their classroom— a major source for stress and burnout (Hirshberg et al., 2020). Compared to more experienced teachers, preservice teachers report lower self-efficacy in their classroom management skills, which has been found to be negatively associated with occupational commitment and positively associated with intentions to leave the profession (Klassen and Chiu, 2011). Many pre-service teachers also cite the lack of student discipline and classroom management skills as a reason for why their teaching may not go well (Brackenreed and Barnett, 2006). Hart (1987) found that the degree of disruption in the classroom correlated highly with the level of anxiety of pre-service teachers. The combined negative effects of role conflict and difficulty in classroom management can compound stress and anxiety for pre-service educators and create conditions ripe for the development of imposter thoughts. These job conditions signal the need to examine the extent to which pre-service educators may experience imposter thoughts. Toward this end our first research question is *what is the prevalence of IS among preservice educators?*

### What Is the Impact of Imposter Syndrome?

Clance (1985) first described the phenomenon of feeling like an imposter. The construct of IS includes feeling *perceived fraudulence* (i.e., believing that you are not worthy of your success or position; c.f. Kolligian and Sternberg, 1991) and fear that others will discover you are a fraud and intense fear of failure (Clance, 1985). Others have described the syndrome in terms of the inaccuracy between self-assessed competence and performance and actual performance (Kets de Vries, 2005; Want and Kleitman, 2006). These inaccurate perceptions arise, in part, due to systematic discounting of positive feedback about performance and because individuals may ascribe accomplishments to luck or over-preparation. A substantive consequence of imposter phenomenon is that those experiencing it are unable to attribute their achievement to internal ability, intelligence, or competence (Clance, 1985; Harvey and Katz, 1985).

Perceiving oneself to be an imposter is associated with a host of negative psychological outcomes, including increased anxiety and depression (Clance and O’Toole, 1987), psychological depletion and distress (Henning et al., 1998; Hutchins and Rainbolt, 2016), poor mental health (McClain et al., 2016) and higher work-family conflict (Crawford et al., 2016). Notably, IS can be characterized as inaccurate self-assessed competence; that is, those with IS under-estimate their competence relative to their actual performance. Some have noted that these inaccurate perceptions may prevent high performers from reaching their full potential or enjoying their success (Clance and O’Toole, 1987). We will subsequently explore our second research question, *what is the possible impact of IS on educator well-being?* We hypothesize that imposter phenomenon will be negatively associated with educator emotional well-being *(H1: Imposter phenomenon will be negatively associated with educator well-being).*

### Demographic Factors and Imposter Syndrome?

Prior research suggests IS is more likely to be experienced by individuals who self-identify as members of gender-, racial-, or sexual orientation- minoritized groups. In this section, we discuss each of these affiliations and potential intersections for IS among pre-service educators.

#### Gender

IS was originally conceptualized to describe the experiences of high performing women who had cycles of imposter thoughts involving fear of failure, guilt about success, and difficulty internalizing positive feedback (Clance and O’Toole, 1987). Some have theorized that women may be particularly vulnerable to imposter thoughts because of their relative under representation in certain professions (Hutchins and Rainbolt, 2016). For example, studies have shown that women in management or leadership positions frequently experience imposter thoughts (Clance and O’Toole, 1987). This view is consistent with research on job-gender context which frames the disparities women face as a direct consequence of under-representation (See job-gender context; Willness et al., 2007).

It is unclear the extent to which women will experience relatively higher rates of imposter thoughts in education because they make up the statistical majority of teachers (upward of 70% the total k-12 teacher workforce; National Center for Educational Statistics [NCES], 2021). Nevertheless, given the wealth of IS research showing women experience high rates of imposter thoughts, we hypothesize that: *H2: Female educators will experience more imposter syndrome compared to male educators*

#### Race

Persons with minortized racial identities are another group for which additional research on imposter syndrome is needed. Drawing from a relational demography perspective (Tsui et al., 1992; Riordan, 2000), some have theorized that persons who identity as members of racially minorized groups may be at increased risk of imposter thoughts because of their underrepresentation in education (only 7% identify as Black, 9% as Hispanic, 2% as Asian, and 2% as mixed-race, whereas 79% of teachers in public schools are white; National Center for Educational Statistics [NCES], 2021). However, the negative effects of being racially different in workgroups are actually stronger and more negative for white (majority group) members than for educators from racially minorized groups (Tsui et al., 1992; Chattopadhyay et al., 2004; Stewart and Garcia-Prieto, 2008). Generally, research shows that racial minorities are less reactive to demographic dissimilarity in their workgroups (Tsui et al., 1992; Chattopadhyay et al., 2004; Stewart and Garcia-Prieto, 2008). As a result, it is presently unclear if and to what extent racial minorities might experience increased IS (relative to majority group members) as a function of their underrepresentation. Additional literature has theorized that minoritized teachers and academics may experience increased imposter thoughts because of stereotypes which threaten their perceptions of competence (i.e., stereotype threat; Edwards, 2019). Examining IS among minoritized racial groups could help explain why educators who self-identify as members of minoritized racial groups are less likely to enter the teaching profession than their peers. We hypothesize that: *H3: Educators belonging to a racial minority will experience more imposter syndrome compared to white educators.*

#### Queer Identities

Relatively little research to date has examined the specific challenges gay, lesbian, bisexual, and queer educators face as a minoritized group in education, and more specifically, how imposter thoughts may affect them. Unlike gender and race, there is little information on the proportion of educators who identify as queer. However, queer individuals make up less than 5% of the total US population (Newport, 2018) and may therefore experience IS in workplaces where they make up a small minority. Furthermore, research on queer educators shows that they often feel unsupported at work and also perceive their workplaces as unsafe (Smith et al., 2008; Wright and Smith, 2015) which may exacerbate the rates at which they feel like imposters. *H4: Queer educators will experience more imposter thoughts compared to heterosexual educators.*

#### Intersectional Identities

Intersectionality refers to the bundles of demographic attributes that generate emergent social identities (Crenshaw, 1989; Liu et al., 2019). Intersectional perspectives take into account that demographic identities may interact and present emergent experiences, including relative positions of advantage/disadvantage and marginalization (Purdie-Vaughns and Eibach, 2008). For example, intersectional research has documented the unique experiences and challenges faced by Black women (Crenshaw, 1989, 1991) noting that they may experience “double jeopardy” in the workplace (Berdahl and Moore, 2006) due to being both female and black. Others have explored the intersection between race and queer identity noting that queer racial minorities may face “intersectional invisibility” (Purdie-Vaughns and Eibach, 2008) – the marginalization experienced because one does not fit the prototype for their racial or queer in-group. As a whole, research on intersectionality has explored the inequities in compensation and experiences of discrimination withing organizations (Liu et al., 2019). Given that research has revealed the unique challenges intersectional individuals face, in this study we will test for interaction effects between race and gender, and race and queer identities on the experiences of IS. We hypothesize that these identities will interact and produce more intense experiences of IS for intersectional minorities. *H5a: Race and gender identities will interact such that female racial minorities will experience more IS than non-female non-racial minorities; and H5b: Race and queer identities will interact such that queer racial minorities will experience more IS than non-queer non-racial minorities.*

### Mitigating Imposter Syndrome: The Role of Emotion Competence

Given prior research has demonstrated both the high rates of imposter thoughts among educators and that IS has deleterious psychological effects on psychological well-being (Clance and O’Toole, 1987; Henning et al., 1998; Hutchins and Rainbolt, 2016; McClain et al., 2016), examining interventions that can mitigate IS feelings is warranted. In this study, we examine the role of emotion competence in mitigating IS. Prior literature has conceptualized theoretically distinct, but overlapping constructs describing emotion competence. Primarily, we consider both “emotion regulation” and “emotion coping” in our theoretical framework of emotion competence. Emotion regulation is defined as “the process by which individuals influence which emotions they have, when they have them, and how they experience emotions” (Gross, 1998). Emotion regulation includes regulating emotions both under normal life conditions and, as is the case for pre-service educators, under conditions of stress. Furthermore, emotional regulation includes both conscious effortful regulation and unconscious regulation. Comparatively coping is defined as “conscious and volitional efforts to regulate emotions, cognition, behavior, physiology, and the environment in response to stressful events” (Compas et al., 2001).

Notably, coping can be considered a “special case” of emotion regulation because it involves conscious emotion regulation under stress (Eisenberg et al., 2010; Compas et al., 2014) whereas emotion regulation might also occur under non-stressful conditions or occur unconsciously (Gross, 2013). Importantly, emotion regulation and coping are related but distinct constructs (Compas et al., 2014) that both capture aspects of effortful and skillful management of thoughts and emotions which may be important for mitigating the impact of imposter syndrome. We investigate both of these constructs because their measures capture different aspects of emotional competence.

In particular, emotional coping (which we measure with the COPE; Carver, 1997) captures the *use* of both adaptive and maladaptive strategies. Research has shown that adaptive strategies are negatively associated with IS, while maladaptive strategies have been found to be positively associated with imposter thoughts. Adaptive coping strategies include positive reframing, acceptance, and seeking support (Carver, 1997; Aldao et al., 2010; Naragon-Gainey et al., 2017). Maladaptive strategies include denial, self-blame, and substance use (Carver, 1997; Aldao et al., 2010; Naragon-Gainey et al., 2017). This comports with broader findings in the literature showing adaptive regulation strategies are protective while maladaptive strategies are harmful (Aldao et al., 2010; Naragon-Gainey et al., 2017).

Likewise, emotion regulation, captures the ability to manage emotions effectively by *applying* appropriate strategies (Aldao et al., 2010). We measure emotional regulation using the most widely cited measure of emotional intelligence (EI), the MSCEIT (Mayer et al., 2003). Emotional intelligence consists of the ability to perceive, understand, use and regulate emotions (Mayer et al., 2003) and in this study we examine the latter facet of emotion regulation. The emotion management measure of the MSCEIT captures the extent to which an individual can judge the effectiveness of strategies in a given situation. Thus, while our measure of coping captures what strategies people typically use, our measure of emotion regulation captures ability in choosing effective strategies— both essential components of emotion competence— and prior research has noted the potential benefit of measuring both of these aspects of emotion competence (Compas et al., 2014).

In this study we examine emotion competence and hypothesize that: *H6: Emotion regulation ability will be negatively associated with imposter thoughts; H7: Adaptive strategy use will be negatively associated with imposter thoughts; and H8: Maladaptive strategy use will be positively associated with imposter thoughts.* Prior research has also demonstrated that emotion regulation can lessen the impact of stress, negative thoughts, emotions and events (Mayer et al., 2008; Gao et al., 2013; Szczygiel and Mikolajczak, 2018). We hypothesize that:*H9: Emotion regulation will moderate and buffer the negative association between imposter thoughts and educator wellbeing.*

## Methods

This study examines the prevalence of IS in a large sample of pre-service educators. Using a survey questionnaire administered prior to an online training, educators reported their feelings of IS as well as their feelings of positive and negative well-being, and measures of emotion regulation and coping strategy use.

### Sample

Participants in this study were recruited through a large teaching organization in the United States which trains and prepares educators for the workforce, Teach for America (TFA). TFA is a non-profit which focuses on developing educators to work in low-income communities across the United States. TFA Corp members typically commit to teaching in low-income communities for 2 years.^1^

3,147 incoming educators were surveyed in June and July of 2020. We note that this survey occurred during the COVID pandemic surge of 2020 and also during the movement for racial justice in the US sparked by the murder of George Floyd. The sample consisted of all pre-service educators, which means that they had not yet taught in a classroom. During the months of June and July, all TFA educators participated in an online training program developed by TFA to prepare them for the classroom in the Fall of 2020. Typically this training occurs in-person; however, due to the pandemic, this training was instead conducted online through webinars, group meetings, and discussions. During this training period, we administered our survey via an online link. Participation in the study was completely voluntary. A total of 1,643 individuals completed the survey and were included in the analysis. This is a completion rate of 52.2%. The sample was on average 24.9 years old (SD = 6.0) and 75.4% female with 1.8% identifying as other or not indicating their gender. The sample identified as 13.9% African American, 7.1% Asian, 15.8% Hispanic, 8.0% Multiracial, 0.6% Native/Indigenous, and 51.6% White. For sexual orientation, the sample identified as 8.4% bisexual, 2.6% gay, 1.5% lesbian, 3.4% queer, and 69.4% straight; 2.3% identified as questioning or other, 12.4% choose not to answer.

To examine the representativeness of our sample in comparison to the population of 3,147 TFA corp members, we conducted supplemental analyses for response bias. Results show no evidence of response bias based on sexual orientation, but some evidence of response bias based on race and gender. The sample included in this study had significantly fewer people of color (53.6% in total TFA corp population vs. 48.4% in our sample; *p* < 0.01), and significantly more women and non-binary individuals (75.8% in total TFA corp population vs. 77.2% in our sample; *p* < 0.05) in comparison to the full population of 3,147 educators.

### Measures

Study measures included Imposter Syndrome, wellbeing, emotion regulation competence, and coping strategy use.

#### Imposter Syndrome

The Clance Imposter Phenomenon Scale (Clance and Imes, 1978) was adapted for an educator population, wherein, the instructions in each item were modified for teaching in this study. Participants were asked to rate on how true the statements were in each item. Examples are, “I have often succeeded on a test or task related to teaching even though I was afraid that I would not do well before I undertook the task,” “I can give the impression that I’m more competent than I really am as a teacher.” Responses were given on a five-point Likert scale from 1 (*Not at all true*) to 5 (*Very true*). A total score was calculated for the 20 items to represent imposter syndrome of each participant; scores on the IS scale range from 20 to 100. Based on definitions provided by Clance and Imes (1978), imposter scores were broken into 4 categories: A score between 41 and 60 was defined as moderate imposter phenomenon experiences; a score between 61 and 80 was defined as frequent impostor syndrome; and a score higher than 80 means frequent intense impostor phenomenon experiences. Scores below 41 were considered not to meet the threshold of imposter syndrome. The internal consistency reliability of the scale was 0.92.

#### Well-Being

Participants were asked to indicate how frequently they have experienced positive and negative emotions over the past few weeks. We used five emotions to measure positive emotional well-being (Thankful, Inspired, Motivated, Excited, Fulfilled) and five emotions to measure negative emotional well-being (Lonely, Disconnected, Exhausted, Worried, Depressed). Responses were given on a five-point Likert scale from 1 (*Not at all*) to 5 (*Always*). Mean scores were calculated for both positive and negative well-being by averaging the responses to the 5 positive well-being items and 5 negative wellbeing items, respectively. Because we took an average of the item responses, wellbeing scores have a range of 1–5. The internal consistency reliability was 0.94 for the positive score, and 0.90 for the negative score.

#### Emotion Regulation Ability

Emotion management ability was measured by the Mayer-Salovey-Caruso Emotional Intelligence Test V2.0 (MSCEIT; Mayer et al., 2003), a commercial EI test. Participants were presented with only the Branch 4, which measures emotion regulation. The items are divided into two parts: emotion management and emotional relationships tasks. In emotion management, participants were asked to rate on how effective the given actions would be in in obtaining the specified emotional outcome for an individual in a story. In the emotion relationships task, participants were asked to rate how effective the actions are for one person to use in the management of another person’s feelings (MSCEIT; Mayer et al., 2003). Responses were given on a five-point Likert scale from 1 (*Very Ineffective*) to 5 (*Very Effective*). The MSCEIT uses proprietary scoring provided by Multi-Health Systems (MSCEIT; Mayer et al., 2003), and scores range from 0 to 1. The internal consistency reliability of the scale was 0.80.

#### Strategy Use

To measure strategy use, we used the brief COPE scale (Carver, 1997). The COPE taps 14 separate emotion coping mechanisms: self-distraction, active coping, denial, substance use, emotional support, instrumental support, behavioral disengagement, venting, positive reframing, planning, humor, acceptance, religion, and self-blame. A self-distraction example item is, “I have been turning to work or other activities to take my mind off of things.” Responses were given on a four-point Likert scale from 1 (I haven’t been doing this at all) to 4 (I have been doing this a lot). We formed a maladaptive coping composite (self-distraction, denial, substance use, behavioral disengagement, venting, self-blame; α = 0.72) and an adaptive coping composite (active coping, emotional support, instrumental support, positive reframing, planning, humor, acceptance, religion; α = 0.79) by calculating the sum of the respective items.

#### Analytic Plan

To test the relationships between IS, well-being, emotion regulation and coping, we examined correlations between these constructs. To test differences between demographic groups on IS, we compared group means on the Clance scale with ANOVA (Tabachnick and Fidell, 2013). Finally, to test if emotion regulation buffers the effect of IS, we regressed well-being onto IS, emotion regulation ability scores and their interaction.

## Results

We organize our results by research question and hypotheses, respectively.

### What Is the Prevalence of Imposter Syndrome Among Pre-service Educators (RQ1)?

We found that 93.4% of the sample experienced at least some level of imposter thoughts; 38.8% had moderate imposter thoughts; 41.9% had frequent imposter thoughts, and 12.7% of the sample experienced severe imposter thoughts (See Table 1).

**TABLE 1:** The prevalence of imposter thoughts.

Imposter thought band	Imposter thought score	*N*	*%*
Below threshold for IP	(0, 40]	109	6.6
Moderate IP	(40, 60]	637	38.8
Frequent IP	(60, 80]	689	41.9
Intense IP	(80, 100]	208	12.7

*Table 1 shows the number and percent of educators in our sample who are considered to have imposter syndrome. The imposter syndrome scale by Clance and Imes (1978) has 4 score bands based on the total score for 20 items which ranges from 20 to 100: A score between 41 and 60 is defined as moderate imposter phenomenon experiences; a score between 61 and 80 is defined as frequent impostor syndrome; and a score higher than 80 means frequent intense impostor phenomenon experiences. Scores below 41 were considered not to meet the threshold of imposter syndrome.*

### What Is the Association of Imposter Syndrome With Educator Well-Being (RQ2)?

We first examined the relationship between IS and well-being. In support of Hypothesis 1, IS scores were negatively correlated with positive well-being (*r* = −0.23, *p* < 0.001) and positively correlated with negative well-being (*r* = 0.43, *p* < 0.001; See Table 2).

**TABLE 2:** Correlation matrix with reliability and descriptive statistics.

	α	M	SD	1	2	3	4	5	6	7	8	9	10	11	12	13	14	15	16	17	18	19	20
1. Imposter thoughts	0.92	62.70	14.9	1																			
2. Positive wellbeing	0.94	3.52	0.68	−0.23*	1																		
3. Negative wellbeing	0.90	2.96	0.69	0.43*	−0.41*	1																	
4. Emotional regulation	0.80	0.39	0.06	−0.05*	0.16*	−0.10*	1																
5. Adaptive coping	0.79	3.20	0.54	−0.09*	0.42*	−0.11*	0.15*	1															
6. Maladaptive coping	0.72	2.24	0.50	0.44*	−0.22*	0.55*	−0.15*	0.06*	1														
7. Self-distraction	0.33	3.31	0.86	0.20*	−0.01	0.22*	−0.01	0.08*	0.47*	1													
8. Active coping	0.71	3.46	0.83	−0.15*	0.35*	−0.20*	0.11*	0.62*	−0.07*	0.02	1												
9. Denial	0.71	1.52	0.78	0.20*	−0.09*	0.28*	−0.17*	−0.01	0.61*	0.16*	−0.50*	1											
10. Substance use	0.95	1.54	0.88	0.12*	−0.12*	0.23*	−0.10*	0.01	0.55*	0.11*	−0.04	0.19*	1										
11. Emotional support	0.80	3.43	0.95	−0.08*	0.30*	−0.11*	0.13*	−64*	0.02	0.03	0.29*	−0.05*	0.02	1									
12. Instrumental support	0.81	3.17	0.98	−0.02	0.26*	−0.03	0.11*	0.67*	0.07*	0.07*	0.30*	−0.02	−0.03	0.69*	1								
13. Behavioral disengagement	0.66	1.61	0.77	0.30*	−0.29*	0.46*	−0.20*	−0.17*	0.63*	0.12*	−0.26*	0.40*	0.21*	−0.15*	−0.10*	1							
14. Venting	0.45	2.74	0.82	0.12*	−0.07*	0.19*	−0.01	0.30*	0.48*	0.06*	0.15*	0.17*	0.14*	0.31*	0.31*	0.14*	1						
15. Positive reframing	0.74	3.50	0.90	−0.09*	0.41*	−0.16*	0.19*	0.64*	−0.04	0.06*	0.35*	−0.04	−0.02	0.30*	0.27*	−0.16*	0.07*	1					
16. Planning	0.68	3.44	0.84	−0.05*	0.28*	−0.05*	0.13*	0.68*	0.04	0.03	0.58*	−0.01	−0.01	0.27*	0.37*	−0.13*	0.16*	0.44*	1				
17. Humor	0.83	2.71	1.15	.13*	−0.01	0.10*	−0.02	0.36*	0.24*	0.12*	−0.03	0.08*	0.19*	0.10*	0.09*	0.11*	0.20*	0.12*	0.06*	1			
18. Acceptance	0.58	3.48	0.82	0.05	0.12*	0.05*	0.06*	0.40*	0.05*	0.06*	0.18*	−0.06*	0.05*	0.14*	0.11*	−0.03	0.14*	0.22*	0.24*	0.13*	1		
19. Religion	0.85	2.36	1.30	−0.19*	0.23*	−0.10*	0.03	0.49*	−0.05*	−0.03	0.23*	0.05*	−0.11*	0.11*	0.16*	−0.05*	0.04	0.25*	0.22*	−0.04	−0.01	1	
20. Self-blame	0.76	2.74	1.10	0.51*	−0.17*	0.48*	−0.06*	−0.01	0.71*	0.19*	−0.07*	0.28*	0.25*	−0.07*	0.02	0.38*	0.18*	−0.06*	0.06*	0.13*	0.02	−0.07*	1

*p < 0.05.

### Who Experiences Imposter Syndrome the Most? Does IS Prevalence Vary as a Function of Minority Identities (RQ3)?

To test if there were differences among minoritized group membership in the severity of imposter thoughts, we conducted a three-way ANOVA on racial identity, gender identity, and sexual orientation with post-hoc follow up comparisons. Table 3 shows the means and SD for all demographic groups compared. When we compared men and women, we found that women had significantly higher imposter thoughts (*M* = 63.33, SD = 15.05) compared to men (*M* = 61.17, SD = 14.58; *F*(1, 1392) = 7.57, *p* < 0.01). These findings support Hypothesis 2. A small portion of our sample (*n* = 13) identified their gender as other or non-binary, and their mean imposter thoughts was 58.69 (SD = 14.93). Due to the small sample size, there was insufficient power to compare non-binary educators to binary educators. Nevertheless, we note that they on average experienced less imposter thoughts compared to their binary peers.

**TABLE 3:** The prevalence of imposter thoughts in different groups.

Group	Imposter thoughts	SD	*F*	*df*	η2
Gender			7.57**	1392	0.01
Women	63.33	15.05			
Men	61.17	14.58			
Race			10.21**	1392	0.03
African American	56.86	15.38			
Asian	63.74	13.71			
White	64.28	14.39			
Hispanic	63.15	15.99			
Multiracial	62.24	14.66			
Sexual Orientation			20.63**	1392	0.01
Gay/Lesbian/Bisexual/Queer	66.34	15.05			
Straight	61.98	14.83			

*p < 0.05, **p < 0.01.

*Table 1 shows the average imposter thought score by demographic group membership. The imposter syndrome scale by Clance and Imes (1978) ranges from 20 to 100.*

When we compared racial minority groups, we found that race was significantly related to imposter phenomenon (*F*(4, 1392) = 10.21, *p* < 0.001). Using Tukey post-hoc comparisons, we found that Black educators (*M* = 56.86, SD = 15.38) had significantly lower imposter thoughts compared to Asian (*M* = 63.74, SD = 13.71), White (*M* = 64.28, SD = 14.39), Hispanic (*M* = 63.15, SD = 15.99) or Multiracial Educators (*M* = 62.24, SD = 14.66). No other group comparisons were significantly different. These results are in contrast to Hypothesis 3 and demonstrate that minoritized educators actually had similar or even lower levels of imposter syndrome compared to White educators in our sample. A small portion of our sample (*n* = 12) identified as native or indigenous, and their mean imposter thoughts was 60.42 (SD = 7.12). Because of the small sample size, we did not have enough power to compare native/indigenous people in our sample, but it is worth mentioning that they on average experienced less imposter thoughts compared to Asian, White, Hispanic, and Multiracial educators.

When we compared educators who identified as straight/heterosexual to those who identified as queer (gay, lesbian, bisexual, questioning, other, or queer), we found that queer (*M* = 66.34, SD = 15.05) reported significantly higher imposter thoughts compared to straight educators (*M* = 61.98, SD = 14.83; *F*(1, 1392) = 20.63, *p* < 0.001), supporting Hypothesis 4.

To test if intersectional identities increased imposter thoughts among educators, we compared the interactions between race, gender, and queer versus straight/cis identities. We did not find a significant interaction between race and gender (*F*(4, 1392) = 0.41, *p* = 0.80), race and queer identity (*F*(4, 1392) = 0.48, *p* = 0.75), or gender and queer identity (*F*(1, 1392) = 0.19, *p* = 0.66), or the three-way interaction (*F*(4, 1392) = 0.64, *p = 0.*63). These results do not support Hypothesis 5a or 5b.^2^

### What Association Does Emotion Regulation Competency Have With Imposter Syndrome (RQ4)?

We found emotion regulation ability had a small negative correlation with imposter thoughts (*r* = −0.05, *p* = 0.03), supporting Hypothesis 6. We also found that adaptive coping strategies had small negative correlations with imposter thoughts (*r* = −0.09, *p* < 0.001). Specifically, active coping (*r* = −0.15, *p* < 0.001), seeking emotional support (*r* = −0.08, *p* = 0.002), reframing (*r* = −0.09, *p* < 0.001), planning (*r* = −0.05, *p* = 0.04), and use of religion (*r* = −0.19, *p* < 0.001) are significantly negatively correlated with imposter thoughts. Whereas imposter thoughts was positively correlated with maladaptive coping (*r* = 0.44, *p* < 0.001). Specifically, distraction (*r* = 0.20, *p* < 0.001), denial (*r* = 0.20, *p* < 0.001), substance use (*r* = 0.12, *p* < 0.001), disengagement (*r* = 0.30, *p* < 0.001), venting (*r* = 0.12, *p* < 0.001) and self-blame (*r* = 0.51, *p* < 0.001) are significantly correlated with imposter thoughts. Seeking instrumental support and acceptance were not significantly related to imposter thoughts. These results support Hypothesis 7 and Hypothesis 8.

To determine if emotion regulation competence buffered the negative associations of imposter phenomenon, we used hierarchical linear regression. We entered the main effects of IS emotion regulation ability as well as their interaction. For the model predicting positive well-being, we found that there was only a main effect of emotion regulation ability and no significant interaction (See Table 4). Emotion regulation ability was positively associated with positive well-being (β = 0.20, *p* = 0.04). Notably, imposter syndrome was not significantly related to positive well-being in the model with the other predictors entered. For negative well-being we found only a significant main effect of imposter thoughts (β = 0.38, *p* = 0.02). Emotion regulation ability did not significantly reduce negative well-being and the interaction between emotion regulation ability and imposter syndrome was also not significant; thus Hypothesis 9 was not supported.

**TABLE 4:** Regression coefficients in predicting well-being using imposter syndrome and ER.

	β	*R* ^2^
**Model for positive well-being**		
Imposter syndrome	−0.12	
Emotion regulation ability	0.20*	
Imposter × ER	−0.11	
		0.08
**Model for negative well-being**		
Imposter syndrome	0.38*	
Emotion regulation ability	−0.08	
Imposter × ER	0.05	
		0.19

ER- emotion regulation ability; *p < 0.05.

## Discussion

This study examined the prevalence and impact of imposter syndrome in pre-service educators. Our findings suggest that most pre-service educators experience imposter syndrome (over 93% of our sample had at least moderate levels of imposter thoughts and 54% had frequent or severe levels of imposter thoughts), and that these thoughts are negatively related to educator well-being. Below we discuss key contributions and the implications of our findings.

First, we found that imposter syndrome plays a significant role in how teachers feel. In particular, we find a substantial positive relationship between imposter syndrome and the negative aspects of well-being (accounting for 18% of the variance in educator’s negative emotional experiences). Imposter syndrome also simultaneously had negative relationships with educators’ positive emotional experiences (accounting for 5% of the variance). These findings reveal the insidious nature of feeling like an imposter— it depletes well-being while increases negative emotional experiences. Demonstrating this link in pre-service educators is an important step for understanding the unique challenges this understudied population faces. Prior literature has noted that pre-service and early-career educators face high uncertainty and high workload (Fogarty and Yarrow, 1994; Paquette and Rieg, 2016) while also reporting less self-efficacy in classroom management (Klassen and Chiu, 2011). Our study demonstrates imposter syndrome is a widespread consequence of these challenges and that this phenomenon is pernicious and harmful.

Second, these findings contribute to understanding the role of minority group membership in the experiences of IS among pre-service educators. Our study found a complex pattern of results whereby women and queer minorities experienced more imposter syndrome compared to other groups. Prior research has also, for example, focused on the job-gender context theorizing that because women are underrepresented in certain occupations, they may have more negative experiences. However, women make up a substantial majority (70%) of the educator workforce, yet still experience more intense imposter syndrome compared to male educators.

Contrary to expectations, we found few differences in the intensity of imposter experiences when we compared racial group membership— despite non-white educators being minoritized within their profession [BIPOC make up around 20% of the educator workforce in the United States]. We also found an unexpected trend that Black educators experienced less imposter syndrome compared to all other racial groups [despite only making up 7% of the educator workforce and 14.8% of our sample]. This pattern of results suggests that racial group representation in an occupation may not be the driving force of imposter syndrome. This comports with prior findings demonstrating that persons who are racially minoritized may not react strongly to racial dissimilarity in workgroups compared to White majority group members (Tsui et al., 1992; Chattopadhyay et al., 2004; Stewart and Garcia-Prieto, 2008). Furthermore, because TFA primarily serves low-income schools where a majority of students are BIPOC, White educators in our sample might experience heightened IS. The effects of teacher-student racial incongruence are well-documented (Joshi et al., 2018), and White teachers working in racially diverse classrooms often report lower job satisfaction and higher stress compared to their BIPOC colleagues (Stearns et al., 2014). Thus, one potential explanation of our finding is that Black educators in our sample experiences lower IS because of racial congruence with their prospective students they were training to serve, while White educators may have perceived racial incongruence. Our pattern of results suggest that imposter syndrome differentially affects minority groups with most intense burden of this phenomenon on pre-service educators falling on women and queer minorities.

Our study also examined demographic group membership from an intersectional perspective. Drawing on prior theories of “double jeopardy” and “intersectional invisibility” we tested whether race, gender and queer identity would interact with one another. We did not find evidence that intersectionality affected the experience of imposter thoughts in our limited sample. One possibility is that the nuances of intersectional identities were not adequately captured in our model, or that there are specific intersectional identities for which IS is more impactful, but these effects may be hidden by other intersectional groups. Future research should intentionally oversample educators who have multiple marginalized identities to investigate this potential interaction with IS further and should investigate which specific intersectional identities might have a unique relationship with IS.

Finally, our study demonstrated that emotion regulation plays a role in mitigating the negative influence of imposter syndrome. Emotion regulation also played an important role in restoring educators’ positive emotional experiences. When we looked at specific strategy use, we found that maladaptive coping strategies had the strongest relationships with imposter syndrome; indicating that the use of these strategies may exacerbate underlying feelings of being an imposter. Comparatively, adaptive coping strategies were negatively related to imposter syndrome among educators. We note that many of the effect sizes we found were small but together, these findings show that how educators manage the stress and demands of being pre-service matters to their well-being. Results present an opportunity to build out supports and interventions to scaffold pre-service educators’ toolbox of coping strategies to supporting in the development and use of adaptive, healthy, coping strategies from the beginning of their careers. Educators, for example, could benefit from early career intervention such emotion regulation training, meditation training, or classroom management strategies.

### Limitations and Future Directions

This study provided a first look into the prevalence and consequences of imposter syndrome among pre-service educators. However, the findings are limited due to the cross-sectional nature of our survey design. Because measures were only given at a single time point, we cannot infer causality of the constructs we measured. Future research should investigate whether maladaptive strategy use is truly an antecedent of feeling like an imposter or, alternatively, weather increased feelings of being an imposter lead one to use more maladaptive strategies to cope with these difficult feelings. Furthermore, our study took place during the COVID pandemic surge of 2020 and during the movement for racial justice in the US. These unique events may have influenced the extent to which educators at this time were prone to experience lower wellbeing and higher imposter syndrome, for example. Future research should seek to replicate our findings and use time lagged designs.

One key finding was that minority group membership had a complex relationship with imposter syndrome. Members of minoritized groups did not universally experience more imposter syndrome in our sample as expected. Instead, we found that women and queer minorities experienced more imposter syndrome while those with minoritized racial identities reported little differences in their experiences of IS when compared to the majority identifying peers. In fact, African American educators experienced less IS. Prior literature has noted that the relationship between imposter syndrome and racial minoritized groups status may be complex or dependent on other social demographic factors (Peteet et al., 2015). Notably, having a strong racial or ethnic identity can be associated with higher identification with career and intention to pursue education for that career (Mendoza-Denton et al., 2008; Duffy and Klingaman, 2009). Thus, rather than causing imposter syndrome, highly developed ethnic identity might prevent or mitigate some of the negative aspects of this phenomenon. Research has also shown that imposter syndrome may be a function of the extent to which organizations are predominantly white institutions (PWI; Peteet et al., 2015). White educators had only a slim majority in our sample (51.1%). Thus, one additional reason we may not have seen increased imposter syndrome among the minoritized educators in our sample could be that the organization where we collected the data had a smaller majority of white educators. It could also be the case that white educators in our sample experienced slightly more imposter syndrome because of the racial heterogeneity in their organization; prior research indicates that white employees react negatively to racial diversity and experience less commitment in racially diverse groups (Tsui et al., 1992) whereas those who identify as BIPOC do not. Because the cohort of pre-service educators we surveyed was racially diverse, those who identify as white in our sample may have experienced more imposter syndrome and contributing to shrinking the difference in reported experiences of IS between White and minoritized educators.

Future research should investigate the mechanisms that might better explain differing experiences of IS among minoritized group members. As discussed, prior literature has focused on the representation of minoritized identities in occupations, but our findings do not support this conjecture since women educators made up 75.4% of our sample (and also make up over 70% of all educators nationally) yet experience more imposter syndrome. Future research should investigate the specific barriers pre-service educators face that might explain why women and queer minorities in particular experience more imposter syndrome. Reasons may include work-life conflict since female pre-service educators may be expected to fulfill roles as educators, students (because they must also pursue training, certifications, and advanced degrees) and, potentially, as family caregivers. Likewise, queer educators may face unique challenges including queer erasure and school environments that are openly hostile to queer identities (Lugg, 2016). For example, 99% of students surveyed in the US report hearing disparaging comments about sexual identity or gender identity (GLSEN, 2020). Future research should explore what factors may make women and queer minorities more susceptible to imposter syndrome and what changes may mitigate imposter syndrome for these minoritized groups.

Our study also examined whether intersectional identities might lead to increased experiences of imposter syndrome. In contrast to our hypothesis, we did not find significant interactions between race, gender, and queer identities. Prior work has noted that multiplicative or interactional quantitative approaches may not be able to capture the nuance of intersectional experiences (Bauer, 2014; Liu et al., 2019). We acknowledge this limitation of our work and encourage future research to examine the effects of intersectionality on educator IS using designs that may capture this nuance (e.g., mixed methodology or qualitative methodology; Harper, 2011).

Finally, we found over 93% of our sample experienced some level of imposter syndrome. Future research should build upon this finding to contextualize why education, compared to other professions, has substantially higher rates of individuals choosing to leave the field. Upward of 11% of educators leave the profession after only a year (Ingersoll et al., 2018). Future research should also seek to confirm if our findings hold in the population of pre-service educators in general. As we noted in the introduction, TFA educators may differ from educators in general in some key ways including that they tend to be recruited from elite pools of candidates and tend not to have prior experience teaching, are asked to work in underserved schools, tend to be more racially diverse, and may view teaching as less of a long term career commitment. These differences could affect the extent to which other educators entering the profession experience imposter syndrome and is a limitation of our study. Thus there is a need to disentangle whether the high rates of imposter syndrome seen among our sample are a function of the experience of entering the classroom for the first time (an anxiety provoking experience all preservice educators must face) rather than a function of the structure of the TFA program or the types of applicants who become TFA corps members. Prior research does indicate that all educators may be at significant risk for experiencing imposter syndrome (Clance and O’Toole, 1987; Knights and Clarke, 2014), which points to our findings being generalizable to the population of all educators, rather than a function of our unique sample.

The contribution of persistent and widespread feelings of being an imposter to educator turnover should be more thoroughly investigated. Future research should also seek to explore avenues to reduce or even prevent these feelings from developing in pre-service teachers. For example, limited evidence exist that shows that group goal sharing can increase feelings of competence (Hardy et al., 2022).

In sum, this study demonstrated that imposter syndrome is a widespread phenomenon in a large sample of pre-service educators and found evidence that IS is negatively related to educator well-being. Further, our study found that adaptive coping strategies may help to mitigate imposter syndrome among pre-service educators and may be particularly beneficial for women and queer minorities. It is of paramount importance that educators with minoritized identities are retained and supported to thrive in our school systems toward the end of supporting authentic, sustainable relationships and role-models for students and their families (Cherng and Halpin, 2016; Carver-Thomas, 2018; Egalite and Kisida, 2018). Targeted intervention to support pre-service educators to navigate feelings of IS through emotion regulation skill-building is one pathway toward this end.

## Data Availability Statement

The raw data supporting the conclusions of this article will be made available by the authors, without undue reservation.

## Ethics Statement

The studies involving human participants were reviewed and approved by Yale IRB. Written informed consent for participation was not required for this study in accordance with the national legislation and the institutional requirements.

## Author Contributions

ML: conceptualization, methodology, formal analysis, investigation, and writing – original draft. PL: conceptualization, methodology, formal analysis, and writing – original draft. CC: writing – review and editing and project administration. MB: writing – review and editing and funding acquisition. All authors contributed to the article and approved the submitted version.

## Conflict of Interest

The authors declare that the research was conducted in the absence of any commercial or financial relationships that could be construed as a potential conflict of interest.

## Publisher’s Note

All claims expressed in this article are solely those of the authors and do not necessarily represent those of their affiliated organizations, or those of the publisher, the editors and the reviewers. Any product that may be evaluated in this article, or claim that may be made by its manufacturer, is not guaranteed or endorsed by the publisher.

## Footnotes

1
https://www.teachforamerica.org/

2 Because our data were collected during the height of the COVID-19 Pandemic and the movement for racial justice following the murder of George Floyd, it is possible that educators in our sample belonging to a minority group may have experienced systematic differences in their well-being. To test if there were differences among minoritized group membership in positive and negative well-being, we conducted two three-way ANOVAs on race, gender, and queer identity with post-hoc follow up comparisons. Queer identity (*F*(1, 1384) = 5.43, *p* = 0.02) was significantly related to positive well-being. We also found a significant interaction effect for race and queer identity for positive well-being (*F*(4, 1384) = 3.16, *p* = 0.01). Using Tukey post-hoc comparisons, we found that queer educators (*M* = 3.44, SD = 0.70) had significantly lower positive well-being compared to straight educators (*M* = 3.55, SD = 0.68). Queer Asian (*M* = 3.16, SD = 0.56) and queer White educators (*M* = 3.39, SD = 0.65) had significant lower positive well-being compared to straight/cis African American educators (*M* = 3.68, SD = 0.74). Gender (*F*(1, 1382) = 12.80, *p* < 0.001) and queer identity (*F*(1, 1382) = 25.80, *p* < 0.001) were also significantly related to negative well-being. Using Tukey post-hoc comparisons, we found that female educators (*M* = 2.98, SD = 0.68) had significantly higher negative well-being compared to male educators (*M* = 2.82, SD = 0.74); queer educators (*M* = 3.12, SD = 0.68) had significantly higher negative well-being compared to straight/cis educators (*M* = 2.90, SD = 0.69). No significant interactions were found.
