# Supplementary material for: Imposter Syndrome Among Pre-service Educators and the Importance of Emotion Regulation
Source: Front Psychol. 2022 Jun 30;13:838575. doi: 10.3389/fpsyg.2022.838575 (PMC9280479; doi:10.3389/fpsyg.2022.838575)
Supplement: Supplementary file 1 [file Table_1.docx]

Supplemental: Analysis for non-response

|  | Coefficient | Included percentage (Minority) | Total percentage  (Minority) |
| --- | --- | --- | --- |
| Race | -0.33^**^ | 48.4% | 53.6% |
| Gender | 0.14^*^ | 77.2% | 75.8% |
| Sexual Orientation | -0.05 | 30.6% | 29.4% |
| Notes: We only included observations with completed surveys in our main analysis. To compare respondents to no respondents, we analyzed data inclusion to determine whether there were key differences between those who are included in the analysis and those who are not. We compared the key demographic characteristics of those who completed the surveys and those who failed to complete the surveys using a univariate probit model. Race, gender, and sexual orientation were used to predict whether the corp member responded or not (included cases are coded to 1, while cases not included are set to be 0). Results show that there is no difference on sexual orientation, but there are significant differences on race and gender.  (1) For race, people of color are coded as 1; for gender, non-males are coded as 1, for sexual orientation, queer identities are coded as 1.  (2) ^*^: p < .05, ^**^: p < .01. | | | |

Appendix

Survey measures

**Clance IP Scale**

For each question, please circle the number that best indicates how true the statement is of you. It is best to give the first response that enters your mind rather than dwelling on each statement and thinking about it over and over.

| **Item** | **Not at all true** | **Rarely** | **Sometimes** | **Often** | **Very True** |
| --- | --- | --- | --- | --- | --- |
| 1. I have often succeeded on a test or task even though I was afraid that I would not do well before I undertook the task. |  |  |  |  |  |
| 2. I can give the impression that I’m more competent than I really am. |  |  |  |  |  |
| 3. I avoid evaluations if possible and have a dread of others evaluating me. |  |  |  |  |  |
| 4. When people praise me for something I’ve accomplished, I’m afraid I won’t be able to live up to their expectations of  me in the future. |  |  |  |  |  |
| 5. I sometimes think I obtained my present position or gained my present success because I happened to be in the right  place at the right time or knew the right people. |  |  |  |  |  |
| 6. I’m afraid people important to me may find out that I’m not as capable as they think I am. |  |  |  |  |  |
| 7. I tend to remember the incidents in which I have not done my best more than those times I have done my best. |  |  |  |  |  |
| 8. I rarely do a project or task as well as I’d like to do it. |  |  |  |  |  |
| 9. Sometimes I feel or believe that my success in my life or in my job has been the result of some kind of error. |  |  |  |  |  |
| 10. It’s hard for me to accept compliments or praise about my intelligence or accomplishments. |  |  |  |  |  |
| 11. At times, I feel my success has been due to some kind of luck. |  |  |  |  |  |
| 12. I’m disappointed at times in my present accomplishments and think I should have accomplished much more. |  |  |  |  |  |
| 13. Sometimes I’m afraid others will discover how much knowledge or ability I really lack. |  |  |  |  |  |
| 14. I’m often afraid that I may fail at a new assignment or undertaking even though I generally do well at what I  attempt. |  |  |  |  |  |
| 15. When I’ve succeeded at something and received recognition for my accomplishments, I have doubts that I can keep  repeating that success. |  |  |  |  |  |
| 16. If I receive a great deal of praise and recognition for something I’ve accomplished, I tend to discount the importance  of what I’ve done. |  |  |  |  |  |
| 17. I often compare my ability to those around me and think they may be more intelligent than I am. |  |  |  |  |  |
| 18. I often worry about not succeeding with a project or examination, even though others around me have considerable  confidence that I will do well. |  |  |  |  |  |
| 19. If I’m going to receive a promotion or gain recognition of some kind, I hesitate to tell others until it is an  accomplished fact. |  |  |  |  |  |
| 20. I feel bad and discouraged if I’m not “the best” or at least “very special” in situations that involve achievement. |  |  |  |  |  |

**Well-being**

This scale consists of a number of words that describe different feelings and emotions. Please indicate how frequently you have experienced each feeling and emotion of the past few weeks

1(not at all) to 5 (always)

Positive emotional well-being

1. Thankful
2. Inspired
3. Motivated
4. Excited
5. Fulfilled

Negative emotional well-being

1. Lonely
2. Disconnected
3. Exhausted
4. Worried
5. Depressed

**COPE scale- Emotion strategy use**

These items deal with ways you've been coping with the stress in your life. For each item, please indicate to what extent you have been doing what the item says.

 1 = I haven't been doing this at all

 2 = I've been doing this a little bit

 3 = I've been doing this a medium amount

 4 = I've been doing this a lot

1.  I've been turning to work or other activities to take my mind off things.

2.  I've been concentrating my efforts on doing something about the situation I'm in.

3.  I've been saying to myself "this isn't real.".

4.  I've been using alcohol or other drugs to make myself feel better.

5.  I've been getting emotional support from others.

6.  I've been giving up trying to deal with it.

7.  I've been taking action to try to make the situation better.

8.  I've been refusing to believe that it has happened.

9.  I've been saying things to let my unpleasant feelings escape.

10.  I’ve been getting help and advice from other people.

11.  I've been using alcohol or other drugs to help me get through it.

12.  I've been trying to see it in a different light, to make it seem more positive.

13.  I’ve been criticizing myself.

14.  I've been trying to come up with a strategy about what to do.

15.  I've been getting comfort and understanding from someone.

16.  I've been giving up the attempt to cope.

17.  I've been looking for something good in what is happening.

18.  I've been making jokes about it.

19.  I've been doing something to think about it less, such as going to movies,

 watching TV, reading, daydreaming, sleeping, or shopping.

20.  I've been accepting the reality of the fact that it has happened.

21.  I've been expressing my negative feelings.

22.  I've been trying to find comfort in my religion or spiritual beliefs.

23.  I’ve been trying to get advice or help from other people about what to do.

24.  I've been learning to live with it.

25.  I've been thinking hard about what steps to take.

26.  I’ve been blaming myself for things that happened.

27.  I've been praying or meditating.

28.  I've been making fun of the situation.
